# Metabolomic Markers Reveal How hCG–Ketoprofen Intervention Increase Pregnancy Percentage Following Timed Artificial Insemination in Dairy Cows

**DOI:** 10.3390/ani16020343

**Published:** 2026-01-22

**Authors:** Hubdar Ali Kolachi, Muhammad Shahzad, Jesse Oluwaseun Ayantoye, Baigao Yang, Xiaomeng Zhang, Pengcheng Wan, Xueming Zhao

**Affiliations:** 1 Institute of Animal Sciences (IAS), Chinese Academy of Agricultural Sciences (CAAS), No. 2 Yuanmingyuan Western Road, Haidian District, Beijing 100193, China; 2023y90100005@caas.cn (H.A.K.); 2023y90100031@caas.cn (M.S.); 2023y90100045@caas.cn (J.O.A.); yangbaigao915@163.com (B.Y.); m843103027@outlook.com (X.Z.); 2 Department of Theriogenology, Shaheed Benazir Bhutto University of Veterinary & Animal Sciences, Sakrand 67210, Sindh, Pakistan; 3 State Key Laboratory of Sheep Genetic Improvement and Healthy Breeding, Institute of Animal Husbandry and Veterinary Sciences, Xinjiang Academy of Agricultural and Reclamation Sciences, Shihezi 832000, China

**Keywords:** dairy cows, timed artificial insemination (TAI), human chorionic gonadotrophin (hCG), ketoprofen, metabolomics, anti-inflammatory effect

## Abstract

The objective of this study was to determine if the use of human chorionic gonadotrophin (hCG) and ketoprofen would affect the pregnancy percentages of dairy cows following timed artificial insemination (TAI). This experiment was conducted on a commercial dairy farm in China involving 799 healthy Holstein cows (2–4 parities) between October and December 2024. Cows were randomly assigned to three groups: hCG-3 = treated with (0 d: GnRH, 100 μg; 7–8 d: PGF_2α_, 0.4 mg; 52 h: GnRH, 100 μg; 14–16 h: TAI) + hCG 3 vials (300 µg)/cow on day 7 of TAI; hCG-2 group = TAI + 2 vials (200 µg) hCG/cow on day 7; and hCG+ketoprofen = TAI + 300 µg hCG/cow + ketoprofen 10 mL/cow on days 15 and 16 of TAI. Metabolomic profiling (untargeted and targeted) of 22 pregnant cows was conducted on serum collected on days 17 and 21 post-TAI. Results indicated greater pregnancy percentages in the cows of the hCG+ketoprofen-treated group compared to those in the other two groups (60.1% compared with 49.6% and 41.9%). The cows treated with hCG+ketoprofen had less oxidative stress markers, downregulation of arachidonic acid metabolism and upregulation of glycerophospholipid metabolism on day 17 after TAI, indicating that there was upregulation of tryptophan and serotonergic pathways, increased amino acid metabolism and continued anti-inflammatory effects on day 21 after TAI. These findings were confirmed by evaluation data collected by conducting the targeted metabolomic procedures, as indicated by the greater progesterone and melatonin and lesser 17-estradiol and 21-deoxycortisol concentrations. These findings indicate that combined hCG+ketoprofen administrations following the TAI treatment regimen improve pregnancy percentages in dairy cattle as a result of metabolic and endocrine milieu modulations.

## Simple Summary

This study was conducted to evaluate whether combining human chorionic gonadotrophin (hCG) and ketoprofen can improve pregnancy percentages in dairy cows after timed artificial insemination (TAI). A total of 799 Holstein cows were assigned to three treatment groups: hCG-3, hCG-2 and hCG plus ketoprofen. The cows in the hCG plus ketoprofen group had the highest pregnancy percentage compared with those in the other two groups. The values obtained from metabolomic analysis of the serum collected on days 17 and 21 after TAI indicated reduced oxidative stress and inflammation, altered lipid and amino acid metabolism and hormonal differences in cows of the hCG+ketoprofen group compared with those of the hCG-3 group.

## 1. Introduction

Researchers are currently working on new methods to decrease embryo/fetal demise during gestation in dairy cows when there was artificial insemination or embryo transfer procedures are imposed to control reproduction with the goal of improving genetics for milk production. These methods are based on the coordinated and timely physiological processes that occur in the embryo, uterine and corpus luteum (CL) during early gestation. The goal is to increase the conception rate in dairy cows [1,2]. One of these methods involves developing a new CL or administering progesterone (P_4_) to enhance the function of the CL and/or increase peripheral (P_4_) concentration and, as a consequence, embryonic development. For this reason, Human Chorionic Gonadotropin (hCG) is one of the hormones that has been extensively investigated in cows [3]. Treatment with hCG results in the development of corpora lutea that have enhanced functions during the early luteal phase, with there being induced ovulation occurring throughout the estrous cycle and consequent alterations in follicular wave dynamics to increase the frequency of estrous cycles in which there are three waves of dominant follicular occurrence [4,5,6]. There are also reports where there was no effect of hCG on the pregnancy percentage, even though P_4_ concentrations were greater in cows treated with hCG [7,8,9,10,11].

Using non-steroidal anti-inflammatory medications to extend the period of corpus luteum functionality is one method of increasing the pregnancy percentages of dairy cows. Non-steroidal compounds have been administered to extend the duration of CL functionality before the time of luteolysis onset. This treatment is thought to result in the embryos with developmental delay producing enough interferon-τ (IFN-τ), which is essential for supporting embryonic viability during the early stages of gestation [12]. The IFN-τ is secreted by the conceptus after transport from the oviduct into the uterus. Interferon-τ inhibits the secretion of PGF_2α_ by endometrial cells, which prevents the lysis of the corpus luteum during maternal recognition of pregnancy in cows, at about day 16 after ovulation [13,14]. Therefore, pharmacological strategies that inhibit the synthesis of PGF_2α_ can be utilized to prevent endometrial PGF_2α_ release during days 15 to 17, subsequent to ovulation, a period recognized to be important for these processes to occur, so there is maintenance of the pregnancy [15,16]. Non-steroidal anti-inflammatory drugs (NSAIDs) prevent the luteolytic function from occurring by inhibiting cyclooxygenase enzymes (COX-1 and COX-2), which are essential for the synthesis of PGF_2α_. NSAIDs such as flunixin meglumine, carprofen, ketoprofen and meloxicam are primarily administered because of analgesic properties [17]. These drugs have also been investigated for their potential to enhance fertility by reducing PGF_2α_ release after artificial insemination (AI) and embryo transfer in cows [18,19] or by preventing luteolysis through the inhibition of endometrial PGF_2α_ synthesis on days 15–16 post-insemination [20]. Ketoprofen, a non-steroidal anti-inflammatory drug, inhibits the biosynthesis of COX-1 and COX-2 enzymes. Although there have been fewer studies where there was an evaluation of ketoprofen effects on pregnancy percentages compared to when there was use of flunixin meglumine, ketoprofen has a lesser half-life circulation, a smaller volume of distribution and is rapidly excreted via the kidneys. The use of ketoprofen is generally associated with fewer side effects but tends to be more costly [17].

A useful new technique for assessing the dynamic metabolic response of biological systems to stimuli or modification is the “metabolomics procedures”, which are being utilized to assess metabolic alterations in dairy cows [21,22]. While metabolomics procedures have proven valuable for understanding physiological responses in various biological systems, there is a notable lack of studies where the metabolic profiles and reproductive outcome associations have been evaluated in cattle. In the present study, we investigated the effect of the luteotropic hormone (hCG) and anti-luteolytic (ketoprofen) treatments on pregnancy outcomes and serum metabolomic profiles in dairy cows subjected to a modified Ovsynch treatment regimen. By integrating hormonal modulation with metabolomic profiling, our findings provide novel insights into potential biomarkers and mechanistic pathways associated with greater fertility.

## 2. Materials and Methods

### 2.1. Animals, Housing Conditions and Experimental Materials

We conducted the study between October and December of 2024 on a commercial dairy farm in China. The experiment was conducted according to the recommendations of the Institutional Animal Care and Use Committee of the Chinese Academy of Agricultural Sciences (Approval No. 2024/08/21, date of approval 21 August 2024). The hormones used in this experiment were all purchased from Ningbo Sansheng Pharmaceutical Co., Ltd. (Ningbo, China), and vials containing ketoprofen were purchased from Foshan City Nanhai Eastern Along Pharmaceutical Co., Ltd. (Foshan, China), without any detailed description, and have been approved for use in animal production.

Holstein dairy cows are kept in feedlots indoors and fed concentrate, rice straw, minerals and vitamins. Drinking water was provided ad libitum. A total of 799 Holstein cattle without any puerperal problems (such as retained placenta, uterine infections, ovarian cysts) as determined during examinations were selected for this study.

### 2.2. Experimental Design

**Figure 1:**
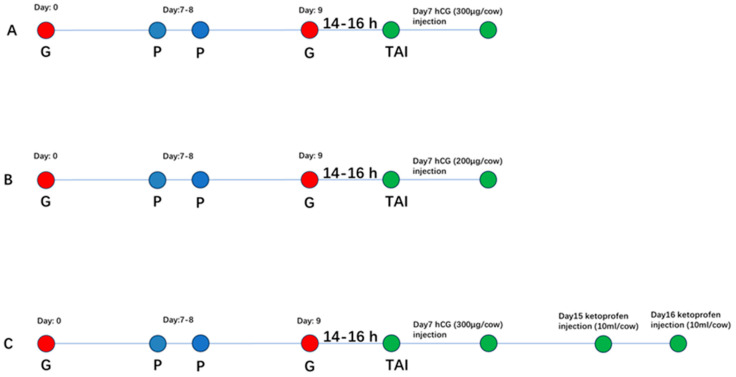
Experimental design for TAI treatment regimens in dairy cows (**A**) hCG-3 group (**B**) hCG-2 (**C**) hCG+ketoprofen group.

The experiment was conducted on 799 dairy cows with a parity status ranging from two to four. Cows were randomly allocated into three treatment groups: hCG-3, hCG-2 and hCG+ketoprofen. All cows followed a standard TAI treatment: 100 μg GnRH on day 0, 0.4 mg PGF2_α_ on days 7–8, 100 μg of GnRH administered 52 h after PGF2_α_, and TAI was performed 14–16 h after the second GnRH injection. Group-specific treatments were as follows: hCG-3 group cows received three vials (300 µg) of hCG/cow on day 7 after TAI (Figure 1A); hCG-2 group cows received two vials (200 μg) of hCG on day 7 after TAI (Figure 1B); and hCG+ketoprofen cows received the same hCG regimen as the hCG-3 group cows with additional ketoprofen (10 mL/cow) administered in two doses on days 15 and 16 of the treatment regimen for conducting TAI (Figure 1C).

### 2.3. Blood Collection and Processing for Metabolomic Analysis

**Figure 2:**
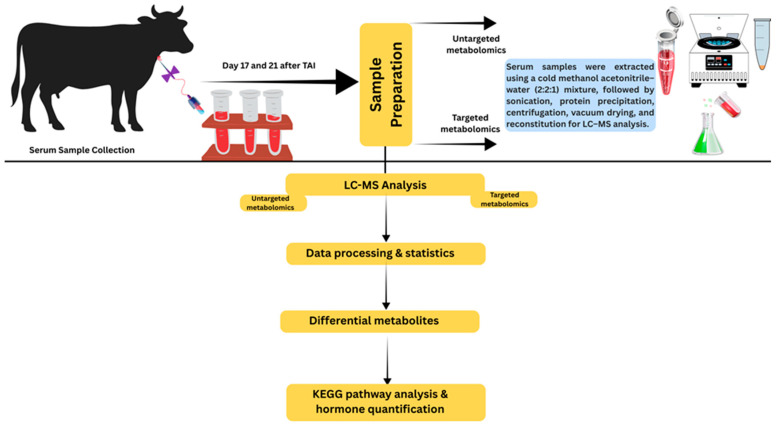
A schematic overview of untargeted metabolomics analysis and targeted metabolite quantification analysis.

For metabolome analysis, blood samples were collected from a subset of dairy cows enrolled in the hCG-3 and hCG-3 plus ketoprofen experiment. To minimize the confounding effect of pregnancy status on circulating metabolites, only cows that were subsequently confirmed pregnant by ultrasonography at day 42 after TAI were included in the metabolomic analysis (*n* = 22 cows). Serum samples of cows diagnosed as non-pregnant were excluded from all metabolomic and pathway analyses. For metabolomic analysis, serum samples were collected at two time points during early pregnancy, day 17 and day 21 after TAI. Serum samples collected on day 17 of the TAI treatment regimen from cows of two groups: (hCG-3 only) and (hCG+ketoprofen) were designated as Group C4 (hCG-3 only) and G4 (hCG-3 plus ketoprofen). Samples collected on day 21 of the TAI were designated as Group C5 (hCG-3 only) and Group G5 (hCG-3 plus ketoprofen). There were 10 mL of blood collected on days 17 and 21 TAI via the jugular vein of each animal using an 18 G needle into a plain (non-anticoagulant) blood collection tube (BD Vacutainer, Franklin Lakes, NJ, USA). The samples were allowed to clot at room temperature for 30 min, then placed on ice and transported to the laboratory. Samples were subsequently centrifuged at 2000× *g* for 15 min at 4 °C. Two 500 μL aliquots of serum were collected and stored at −80 °C for metabolomic analysis. Samples were maintained at −80 °C until further processing. A schematic overview of the experimental workflow, including sample collection, untargeted metabolomics analysis and targeted metabolite quantification, is presented in Figure 2.

### 2.4. Serum Metabolite Analysis

#### 2.4.1. Chromatography-Mass Spectrometry

The samples were first thawed on ice and then extracted using a pre-chilled methanol–acetonitrile–water mixture (2:2:1, *v*/*v*). After thorough vortexing, the extracts were sonicated at low temperature for 30 min and subsequently placed at −20 °C for 10 min to enhance protein precipitation. The mixture was then centrifuged at 14,000× *g* for 20 min at 4 °C. The resulting supernatant was dried under vacuum, and the residues were reconstituted in 100 µL of acetonitrile–water (1:1, *v*/*v*) for LC–MS analysis. The solution was gently mixed, centrifuged again at 14,000× *g* and 4 °C for 15 min, and the clarified supernatant was transferred for instrumental analysis.

The samples were analyzed using an Agilent 1290 Infinity UHPLC system equipped with a HILIC column maintained at 25 °C (Santa Clara, CA, USA), with a flow rate of 0.5 mL/min and an injection volume of 2 µL. The mobile phase consisted of water containing 25 mM ammonium acetate and 25 mM ammonia (phase A) and acetonitrile (phase B). Metabolite separation was achieved using a HILIC gradient program (XCMS 4.0) as follows: 0–0.5 min, 95% B; 0.5–7 min, linear decrease from 95% to 65% B; 7–8 min, linear decrease from 65% to 40% B; 8–9 min, hold at 40% B; 9–9.1 min, increase from 40% to 95% B; and 9.1–12 min, hold at 95% B for column re-equilibration. Throughout the period procedures were being conducted, samples were maintained at 4 °C in the autosampler. To minimize instrument-related variation, all samples were injected in randomized order. The first three QC samples were used to monitor instrument status and equilibrate the chromatographic-mass spectrometric system. The next three QCs were used for segmented scanning to identify metabolites. An AB Triple TOF 6600 mass spectrometer (Framingham, MA, USA) was used for the acquisition of primary and secondary spectra of the samples. The samples were separated by an Agilent 1290 Infinity LC ultra-high performance liquid chromatography system (UHPLC) and then analyzed by mass spectrometry on a Triple TOF 6600 mass spectrometer (AB SCIEX) in electrospray ionization (ESI) positive and negative ion modes, respectively. The ESI source was set up with the following parameters: atomization gas, auxiliary heating gas 1 and 2 were both 60, air curtain gas was 30 psi, and the ion source temperature was 600 °C. Spray voltages of ±5500 V were used for both positive and negative modes. The primary mass-to-charge detection range was 60–1000 Da with a scan accumulation time of 0.20 s/spectra; the secondary daughter ion mass-to-charge detection range was 25–1000 Da with a scan accumulation time of 0.05 s/spectra. The secondary mass spectra were acquired in the data-dependent acquisition mode (IDA) and filtered by the peak intensity values, and the de-clustering voltages (DPs) were as follows: ±60 V (both positive and negative modes), collision energy of 35 ± 15 eV, IDA set to dynamically exclude isotope ions in the range of 4 Da, and 10 fragmentation profiles were acquired per acquisition cycle.

#### 2.4.2. Targeted Quantification of Estradiol, Progesterone, Melatonin and 21-Deoxycortisol

The UHPLCMS/MS procedure was used to determine metabolite quantification in the serum of day 17 samples only through targeted metabolomics. The separation was conducted on a reversed-phase C18 column at 35 °C with water (A) containing 0.1% formic acid and methanol (B) containing 0.1% formic acid at 0.3 mL/min. The injection volume was 5 µL. Multiple reaction monitoring (MRM) was employed to quantify the different MS/MS using the isotopically labeled internal standards (E2-d4 and P4-d9) with high accuracy. The analytical method was validated according to generally accepted bioanalytical guidelines. Calibration curves were made at a minimum of six concentrations and showed good linearity for all analytes (R^2^ > 0.99). Method accuracy and precision were evaluated with quality control samples at relatively lower, medium and relatively greater concentrations, which were included in each analytical batch. Intra- and inter-batch precision values were within the acceptable limits (coefficient of variation < 15%). The limits of detection and quantification were adequate to reliably measure all the target hormones in physiological concentration ranges in the serum of cattle. All serum samples were stored at −80 °C and analyzed under the same conditions to reduce any analytical variation.

### 2.5. Pregnancy Status Evaluation

At 42 days following TAI, a skilled professional determined pregnancy status using a B-ultrasonographic device (DP-30Vet, Shenzhen Mindray Biomedical Electronics Co., Ltd., Shenzhen, China). A distinct embryo and associated embryonic membrane were detected in the B-ultrasonic image if cows were classified as being pregnant. The pregnancy percentages were computed as the number of pregnant cows relative to the total number of cows inseminated.

### 2.6. Data Preprocessing, Analysis and Metabolite Identification

For untargeted metabolomics, first, the raw data from the mass spectrometer were converted into mzXML format using ProteoWizard (3.X) [23]. Peak alignment, retention-time correction and area extraction of peaks were performed using XCMS (v3.7.0). The identification of metabolites was based on an in-house library of standard metabolites. Data preprocessing subsequently occurred, which included structural annotation and quality evaluation, followed by downstream analysis. The criteria of selection of differential metabolites were VIP > 1.0, fold change (FC) < 0.833 or >1.2 and *p* < 0.05. The KEGG analysis was performed to elucidate the pathways associated with differentially abundant metabolites. The fold change threshold is used as a prioritization criterion to emphasize metabolites with greater effect sizes, while pathway enrichment analyses incorporated all statistically significant metabolites (*p* < 0.05), ensuring that biologically relevant changes with modest fold effects were retained. For targeted hormone quantification, estradiol, progesterone, melatonin and 21-deoxycortisol concentrations were calculated from calibration curves using the peak area ratios relative to internal standards. Statistical analysis, including pregnancy percentage comparisons, was performed using GraphPad Prism 10.4.2 (633) with a chi-square test for categorical data.

## 3. Results

### 3.1. Data for the Effects of hCG and Ketoprofen on Pregnancy Percentages as a Result of Conception Resulting from TAI

Pregnancy percentages for cows in the hCG+ketoprofen group were greater than in cows of groups treated with only hCG (*p* < 0.05). These findings indicate treatment with hCG and ketoprofen resulted in greater pregnancy percentages in dairy cows (Table 1).

### 3.2. Metabolite Classification in Cattle After Ketoprofen Treatment

**Figure 3:**
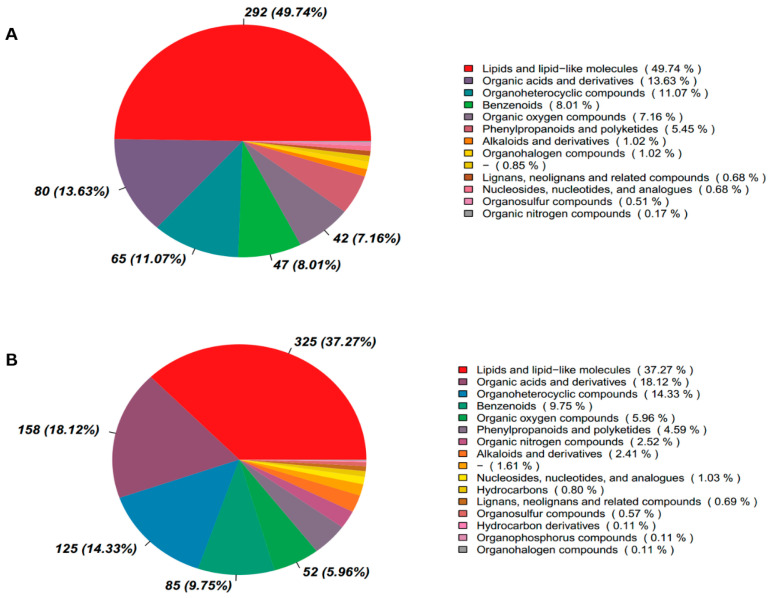
Metabolite Classification in cattle after ketoprofen treatment in cows on days 17 and 21 after TAI (**A**) Negative ion mode (**B**) Positive ion mode. On the right side of the depiction is the proportion of metabolites, and on the left is a pie chart that illustrates the relative distribution (percentage) of identified metabolites among major chemical classes.

To investigate the factors contributing to the increased pregnancy percentages following ketoprofen treatment and determine reasons for the relatively lesser pregnancy percentages in cows of hCG group, serum samples from cows subsequently confirmed pregnant by ultrasonography in both (hCG-3 only) and experimental groups (hCG+ketoprofen) collected on day 17 (C4 and G4) and 21 (C5 and G5) of TAI, were subjected to untargeted metabolomic analysis. In the analysis of serum samples, 872 metabolites were identified in positive ion mode (POS) and 587 in negative ion mode (NEG). Analysis across both modes indicated there was a predominance of lipid and lipid-like molecules, accounting for 37.3% in POS and 49.74% in NEG (Figure 3A,B). Additionally, the KEGG database was used to functionally annotate the detected metabolites and to interpret the biological function and classifications (Supplementary Figure S1A,B). In both modes, these metabolites were involved in pathways associated with transport and catabolic processes, cell growth and apoptosis, signal transduction, membrane transport, lipid and energy metabolism, glycan metabolism and xenobiotic degradation.

### 3.3. Screening of Differentially Abundant Metabolites Associated with Reproduction and Fertility on Day 17 of the TAI Treatment Regimen (Cows of C4 Compared with Those of G4 Group)

**Figure 4:**
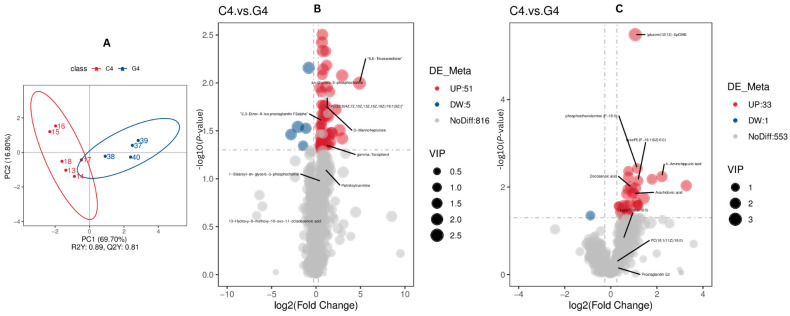
Screening of differential metabolites associated with reproduction and fertility (C4 and G4). The PLS-DA scatter plots for (**A**) combine positive ion and negative ion modes, where the *x*-and *y*-axes represent scores on the first and second principal components. The R^2^Y value indicates model fit, Q^2^Y reflects predictive capacity, and models are robust when R^2^Y > Q^2^Y. Volcano plots for (**B**) positive and (**C**) negative modes depict metabolites where there were no detectable differences (gray), upregulated (red) and downregulated (blue), with VIP values indicating metabolite contribution to group separation.

Partial Least Squares Discriminant Analysis (PLS-DA) was used to determine a relationship model between metabolite abundance and sample categories, allowing for reliable discrimination and prediction of sample classification. To discriminate the differentially abundant metabolites between C4 and G4, a supervised PLS-DA model was utilized. As depicted in Figure 4, the evaluation model yielded R^2^Y values of 0.89 in combined mode, while the corresponding Q^2^ values were 0.81 (Figure 4A). The relatively greater R^2^Y values compared to Q^2^ indicate the model reliability.

For C4 and G4 comparison, there were a total of 90 differentially abundant metabolites in both positive and negative mode (Figure 4B,C). A heat map displaying all differentially abundant metabolites in both modes is provided in Supplementary Figure S2A,B.

**Figure 5:**
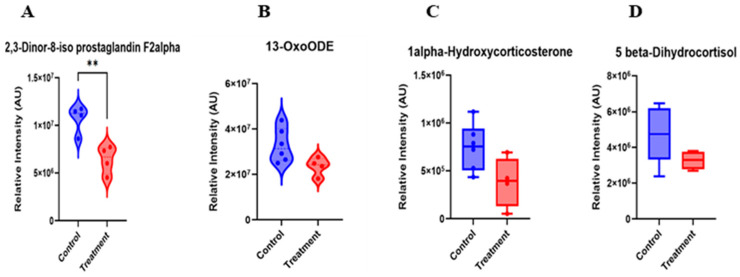
Serum Metabolites-Biomarkers identified on d17 of TAI after ketoprofen treatment. Asterisks indicate statistically significant differences between groups (*p* < 0.01).

Following filtering for metabolites relevant to reproduction and fertility with a fold change (FC) > 1.2, eight differentially abundant metabolites were identified between cows of the C4 and G4 samples (Table 2). Notably, 2,3-Dinor-8-iso PGF_2α_ and 13-OxoODE had relatively lesser intensities in the hCG+keoprofen treatment compared with the hCG-3 group (Figure 5A,B). Thus, the results from treatment of cows with the ketoprofen compound indicate there was mitigation of oxidative damage via neutralizing free radicals and preventing lipid peroxidation. This finding is considered to indicate that the treatment successfully reduces lipid peroxidation and inflammatory processes. Similarly, steroid-related metabolites, including 1α-hydroxycorticosterone and 5β-dihydrocortisol, were downregulated in cows of the treatment group (Figure 5C,D). This pattern suggests a modulation of glucocorticoid metabolism, which may reflect reduced activation of stress-associated endocrine pathways.

### 3.4. The Effects of Ketoprofen on Metabolic Pathways on Day 17 After TAI

**Figure 6:**
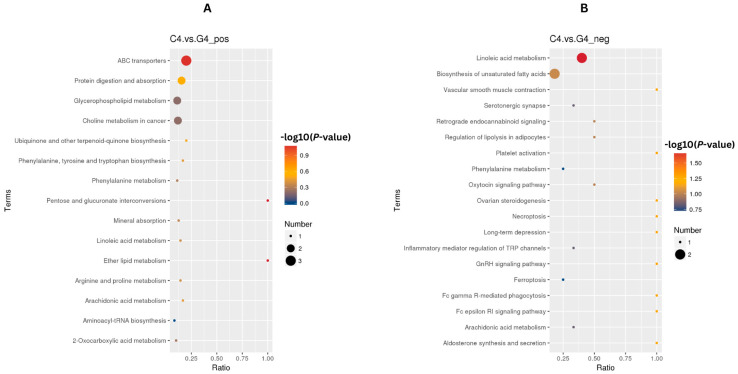
Metabolic pathway changes indicative of ketoprofen’s anti-luteolytic effects. KEGG pathway enrichment analysis of ketoprofen-treated G4 compared with the C4 groups, depicting (**A**) upregulated pathways supporting luteal cell survival and steroidogenic function and (**B**) downregulated pathways preventing PGF_2α_-mediated luteolysis. Dot color represents value differences (−log10 (*p*-value), FDR < 0.05), dot size indicates gene count per pathway and *x*-axis depicts enrichment ratio.

In both POS and NEG ion mode, findings from conducting the KEGG enrichment analysis (FDR < 0.05) following ketoprofen treatment revealed modulation of metabolic pathways. The enriched pathways suggest a coordinated metabolic response characterized by suppression of luteolytic process alongside luteal support. Specifically, pathways associated with the metabolism of arachidonic acid, which is important for PGF_2α_ synthesis, and vascular smooth muscle contraction, related to regulation of luteal blood flow were downregulated in the ketoprofen treated group. These changes are consistent with suppression of luteolytic signaling and maintenance of luteal vascular function. Concurrently, pathways associated ABC)transporters and glycerophospholipid metabolism were also enriched, suggesting increased support of progesterone transport and luteal cell membrane integrity and function. Collectively, these alterations in the pathway level suggest that ketoprofen treatment may contribute to enhanced luteal stability and progesterone supportive metabolic conditions during maternal recognition period for pregnancy and may favor pregnancy maintenance (Figure 6A,B).

### 3.5. Screening of Differentially Abundant Metabolites Associated with Reproduction and Fertility on Day 21 After TAI (C5 Compared with G5)

**Figure 7:**
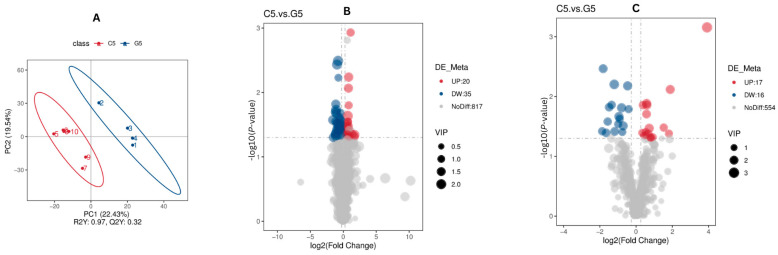
Screening of differentially abundant metabolites associated with reproduction and fertility (C5 compared with G5). The PLS-DA scatter plots for (**A**) combine positive ion and negative ion modes, where the *x*- and *y*-axes represent scores on the first and second principal components. The R^2^Y value indicates model fit, Q^2^Y value reflects predictive capacity and models are robust when R^2^Y > Q^2^Y. Volcano plots for (**B**) positive and (**C**) negative modes indicate metabolites that were not differentially abundant (gray), upregulated (red) and downregulated (blue), with VIP values indicating metabolite contribution to group separation.

To discriminate the differentially abundant metabolites between C5 and G5, a supervised PLS-DA model was utilized. As depicted in Figure 4, the evaluation model yielded R^2^Y values of 0.97 in combined mode, while the corresponding Q^2^ values were 0.32 (Figure 7A). The relatively greater R^2^Y compared with Q^2^ values indicate the model reliably.

For C5 _G5 comparison, a univariate *t*-test analysis was conducted with results indicating there were 88 differentially abundant metabolites in both positive and negative mode (Figure 7B,C). A heat map revealing all differentially abundant metabolites in both ion modes is provided in Supplementary Figure S3A,B.

**Figure 8:**
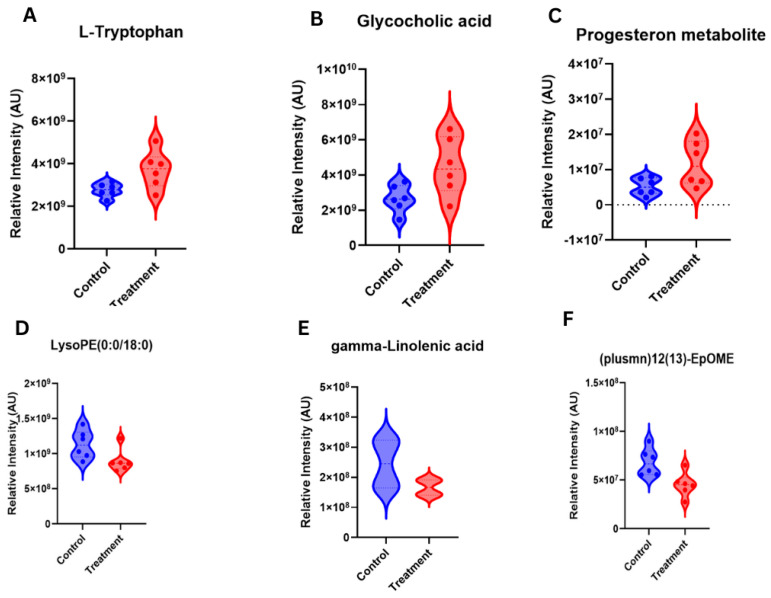
Serum metabolites-biomarkers identified on d21 of TAI after ketoprofen treatment.

Following filtering for metabolites relevant to reproduction and fertility with a fold change (FC) > 1.2, nine differentially abundant metabolites were identified between samples from cows in the C5 and G5 groups (Table 3). Notably, L-Tryptophan, Glycolic acid and 3α,20α-dihydroxy-5β-pregnane glucuronide exhibited relatively greater intensities in the treatment group compared with the hCG-3 group (Figure 8A–C). Conversely, gamma-Linolenic acid, 12(13)-EpOME and LysoPE(20:3) had lesser intensities in the cows of the treatment group compared to the hCG-3 group (Figure 8D–F).

### 3.6. The Effects of Ketoprofen on Metabolic Pathways on Day 21 After TAI

**Figure 9:**
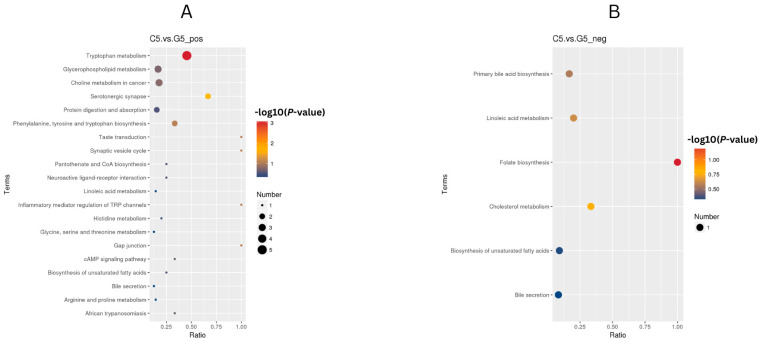
KEGG enrichment analysis of C5 and G5 (**A**) Upregulated and (**B**) downregulated pathways (FDR < 0.05) in G5 versus C5 comparison. Color indicates (−log10 (*p*-value)), size indicates gene count, *x*-axis is indicative of enrichment ratio. Results indicate there was enhanced tryptophan metabolism and serotonergic signaling with suppressed steroid precursor pathways, suggesting coordinated remodeling of amino acid, neuroendocrine and lipid metabolism.

Findings from conducting the KEGG enrichment analysis (FDR < 0.05) of cows in the C5 compared with G5 group on day 21 indicated there were metabolic responses characterized by enhanced neuroendocrine function and amino acid metabolism. Pathway upregulation included tryptophan metabolism, serotonergic synapse pathways and comprehensive amino acid processing (phenylalanine, tyrosine and tryptophan biosynthesis; arginine, proline metabolism), combined with greater cellular communication mechanisms (gap junction, synaptic vesicle cycle). Lipid-associated pathways, including folate biosynthesis, cholesterol metabolism, primary bile acid biosynthesis and biosynthesis of unsaturated fatty acids, were also overrepresented, suggesting the coordinated remodeling of one-carbon and lipid metabolism (Figure 9A,B).

### 3.7. Targeted Metabolomics of Estradiol, Progesterone, Melatonin and 21-Deoxycortisol

**Figure 10:**
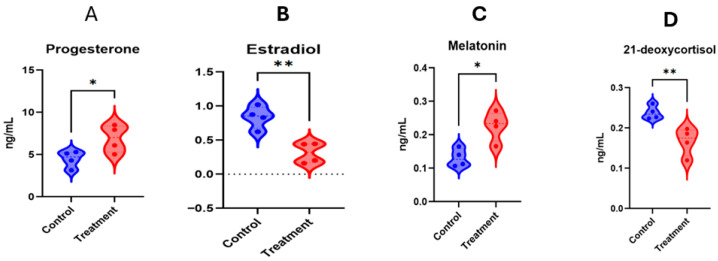
Targeted metabolite quantifications on d17 after TAI in ketoprofen treated cows. Asterisks indicate statistically significant differences between groups * *p* < 0.05; ** *p* < 0.01.

The purpose for conducting the targeted metabolomics procedures was to quantify these important metabolites in the serum at day 17 of TAI. The selection of four metabolites (estradiol, progesterone, melatonin and 21-deoxycortisol) was performed because of physiological implication in luteal functioning, inflammation and pregnancy initiation. Figure 10A,B depicts that progesterone and melatonin concentrations were greater (*p* < 0.05) in the treatment (hCG+ketoprofen) compared with the hCG-3 group, while estradiol and 21-deoxycortisol concentrations being less in the hCG+ketoprofen compared to hCG-3 group (Figure 10C,D).

## 4. Discussion

In the present study there was utilization of metabolomic profiling procedures to gain a greater understanding of the molecular mechanisms underlying improved pregnancy percentage following combined hCG and ketoprofen treatment in dairy cattle after timed artificial insemination. Our findings indicate that this dual hormonal-pharmacological treatment greatly improved the pregnancy percentages in two independent experiments and generated specific metabolic signatures at the crucial time (days 17 and 21) of the maternal recognition of pregnancy window. The results give new insights into the potential mechanisms of how the anti-luteolytic effect of ketoprofen and the luteotropic effect of hCG may interact to enhance embryonic survival in the early embryo by regulating the activity of some critical metabolic pathways.

### 4.1. Effect of hCG and Ketoprofen Following TAI on the Pregnancy Percentage of the Dairy Cows

In this experiment, the hCG and ketoprofen combination treatment showed a significant improvement in the pregnancy rate over the hCG groups (*p* < 0.05). Thus, supporting our hypothesis that a dual intervention strategy of both corpus luteum support and prostaglandin-mediated luteolysis would make a difference in fertility. These results are consistent with other reports that have proven the positive impacts of post-insemination hormonal interventions. Uçar and Peker (2023) found pregnancy percentages of 64% compared with combined hCG and ketoprofen treatment following Ovsynch-based TAI in Holstein cows [24], findings that were similar to those in the present study. The increased pregnancy percentages are likely due to the luteotrophic effect of hCG that leads to relatively greater progesterone concentrations in circulation [4,25] and the ketoprofen that blocks the production of prostaglandins F_2α_, preventing premature luteal degradation during the period of maternal recognition of pregnancy [26,27]. The timeline of the intervention was determined to align with days 4 to 7 after the induction of accessory CL formation and long-term progesterone secretion between day 11 after TAI, when embryonic (IFN-τ) is produced to mitigate endometrial PGF_2a_ release [28], and days 15–16 after TAI, when the inhibition of luteolytic signaling occurs [10,29,30]. Results from a meta-analysis [10] indicated that hCG therapy (≥1500 IU) administered 5 to 7 days following synchronized ovulation induction led to a greater conception percentage following embryo transfer, especially in multiparous lactating cows and those with relatively lesser than expected fertility [11]. By combining these two interventions, there is mitigation of the marginal luteal support (by hCG treatment) and premature luteolysis (by ketoprofen treatment), addressing the major causes of early embryonic demise during the early stages of gestation in dairy cows. Findings in the present study extend previous research on NSAID and hCG use in cattle reproduction by providing the first comprehensive metabolomic characterization of these interventions. There has been no utilization of which we are aware of untargeted metabolomics to identify the molecular markers after imposing treatments in attempts to enhance reproductive success in dairy cows.

### 4.2. Metabolomic Profiling Findings Reveal Distinct Treatment-Induced Biomarker Changes

By conducting the untargeted metabolomics analysis procedures in the present study, there were 872 metabolites that were detected in positive ion mode and 587 metabolites in negative ion mode, and lipid and lipid-like molecules formed the largest class (37.3% in POS ion mode and 49.7% in NEG ion mode). The predominance of lipid metabolites is indicative of important functions in steroid hormone biosynthesis, cell membrane structure, energy metabolism and cell signaling; all of which are important processes in the functioning of the corpus luteum, endometrial receptivity for the embryo and the initiation of embryonic growth [31,32]. The findings regarding this lipid-based metabolic compound are consistent with previous findings in cattle reproduction studies where there was consistent identification of the importance of glycerophospholipid metabolism, fatty acid oxidation and lipid signaling pathways in the establishment of pregnancies and embryo viability [22,33,34]. The data resulting from PLS-DA models indicated there was a good fit (R^2^Y ranging between 0.89 and 0.97) with predictive power (Q^2^ 0.81 and 0.32), suggesting the reliability of the model in both groups. The utilization of univariate analysis procedures led to the determination that there were 90 and 88 differentially abundant metabolites at days 17 and 21 after AI, respectively, thus indicating that there was metabolic remodeling induced by treatment.

### 4.3. Day 17 Metabolomic Biomarkers: Anti-Inflammatory and Anti-Luteolytic Mechanisms

#### 4.3.1. Suppression of Oxidative Stress and Lipid Peroxidation Markers

hCG+ketoprofen treatment significantly reduced the serum concentrations of 2,3-Dinor-8-iso prostaglandin F_2a_ and 13-OxoODE in the cows of the treatment group compared to the hCG treatment group at day 17 after TAI. These metabolites are well-known oxidative stress and lipid peroxidation biomarkers [35,36,37,38]. The reduction in these oxidative stress markers in the cows of the treatment group indicates that ketoprofen has cytoprotective effects in addition to the cyclooxygenase inhibition activity. This results from the greater than optimal concentration of ROS disrupting cellular components, embryo development and endometrial functionality [39,40]. Ketoprofen can potentially provide an enhanced uterine milieu by inhibiting lipid peroxidation, in which embryo implantation and early development can occur. These findings add to the prior understanding of NSAIDs in cattle reproduction, which has been solely concentrated on the inhibition of prostaglandin synthesis [41,42].

#### 4.3.2. Modulation of Arachidonic Acid Metabolism

The KEGG pathway enrichment analysis results in the present study indicate that arachidonic acid metabolism was downregulated in the treatment group at day 17 after TAI, indicating the primary mechanism of action for ketoprofen. Arachidonic acid is a precursor for prostaglandin synthesis via the COX pathway, and PGF_2α_ is the main luteolytic agent in cattle [43,44]. During maternal recognition, the embryo’s IFN-τ must inhibit endometrial PGF_2α_ pulses to prevent CL regression [14]. Ketoprofen is a non-selective COX inhibitor that, when administered, enhances this signal by mitigating the conversion of arachidonic acid to prostaglandin H2 [45] and, in doing so, protects cattle embryos during early developmental stages, because embryos might produce optimal concentrations of enough IFN-τ by day 16 and 17 after AI. Interestingly, in the present study, there was an upregulation of arachidonic acid (FC: 2.02, *p*: 0.014) in the treatment group. This seemingly paradoxical result of more substrate and less pathway activity is presumably an indication of an increase in arachidonic acid concentration resulting from COX inhibition. Inhibition of the arachidonic acid enzymatic conversion results in the substrate accumulation in the cellular pools [46]. These greater-than-optimal arachidonic acid concentrations could have other biological implications because arachidonic acid can also be processed in other pathways (lipoxygenase and the cytochrome P450 pathway) to produce lipoxins, epoxyeicosatrienoic acids (EETs) and other lipid mediators that possess anti-inflammatory and pro-resolution effects [47,48].

#### 4.3.3. Suppression of Cortisol Pathway Metabolites

In the present study, there was downregulation of steroid-related metabolites, such as 1α-hydroxycorticosterone and 5β-dihydrocortisol, suggesting modulation of stress-related endocrine signaling [49,50]. Greater than optimal cortisol concentrations have been linked to reduced fertility in dairy cattle, because chronic stress can disrupt reproductive hormone secretion, impair immune function and compromise embryo receptivity [51,52]. Stress-induced increases in circulating cortisol can suppress the GnRH and LH secretion, resulting in less-than-optimal corpus luteum function and progesterone biosynthesis [53]. In addition, cortisol can directly affect endometrial gene expression, as well as the milieu of the uterus in ways that are harmful to embryo survival [54,55].

#### 4.3.4. Upregulation of Glycerophospholipid Metabolism

A number of glycerophospholipid metabolites were upregulated in the treatment group at day 17 after TAI, such as sn-glycero-3-phosphocholine, PC(18:0/18:1-O(12,13)), LysoPE(P-18:1/0:0) and phosphoethanolamine (P-18:0). Glycerophospholipids are essential membrane constituents that facilitate cellular signaling, membrane transport and lipid metabolism [56]. Up-regulation of these compounds is probably linked to increased biosynthesis and remodeling of membranes in the maintenance of the corpus luteum, especially considering that hCG induces accessory CL formation that leads to extensive massive membrane expansion during luteal cell development [57]. Phosphatidylcholine and phosphatidylethanolamine are precursors of phosphatidylcholine and phosphatidylethanolamine, the most common membrane phospholipids in the mammalian cell [58]. Bioactive mediators that facilitate cell survival and proliferation are also lysophospholipids like LysoPE [59], which may support prolonged luteal functioning and the production of progesterone. Previous metabolomic analyses in cattle have correlated glycerophospholipid profiles with greater pregnancy percentages [60,61]. Findings in the present study extend this evidence with indications that the pharmacological treatment is capable of regulating phospholipid metabolism in such a way that might result in improved reproductive performance.

#### 4.3.5. Kegg Pathway Analysis: Dual Luteotropic and Anti-Luteolytic Mechanisms

There was a dual effect of ketoprofen (KEGG enrichment at day 17) on both luteolytic (arachidonic acid metabolism, vascular smooth muscle contraction) and luteal-support (ABC transporters, glycerophospholipid metabolism) pathways. The downregulation of vascular smooth muscle contractions is remarkable because luteolysis is characterized by a decrease in steroidogenesis as well as a decline in luteal blood flow [62]. Administration of ketoprofen could allow the maintenance of CL and aid progesterone production by modulating vascular tone. Evidence of the upregulation of ABC transporter pathways indicates enhanced cell export potential, which is an essential and crucial phase in the release of steroid hormones. The ABC transporters, including ABCG1 and ABCA1, mediate the transport of cholesterol and steroids in steroidogenic tissues [63,64]; hyperactivity of this process could stimulate the release of progesterone and reinforce the embryonic supporting functions.

### 4.4. Day 21 Metabolomic Signature: Neuroendocrine Optimization and Metabolic Remodeling

#### 4.4.1. Upregulation of Tryptophan and Serotonergic Pathways

L-tryptophan concentrations were greater in the treatment group (FC: 0.74, *p*: 0.038), and results from KEGG pathway analysis indicated tryptophan metabolism was the most enriched pathway that was upregulated along with serotonergic synapse pathways, synaptic vesicle cycle and gap junction signaling. Tryptophan is an essential amino acid, which is the precursor for the biosynthesis of serotonin (5-hydroxytrytomine, 5-HT) [65]. Serotonin is traditionally known as a neurotransmitter in the central nervous system, but it is also an important signaling molecule in peripheral tissues, such as the reproductive tract [66,67]. In the uterus, serotonin regulates vascular tone, smooth muscle contractility, immune function and decidualization [68]. The enhanced tryptophan metabolism and serotonergic activity at day 21 after TAI is indicative of the shift in direct anti-luteolytic effects (dominant at day 17) to longer-term reproductive support via neuroendocrine optimization. By day 21 after TAI, maternal recognition of pregnancy (MRP) occurs, and there is a subsequent induction of physiological and endocrine processes for maintaining an optimal uterine milieu for continued embryonic development and implantation [69]. The vascular regulation and immune modulation functions of serotonin might also be a part of this process because these could enhance endometrial embryonic receptivity and control local inflammatory processes [70,71].

#### 4.4.2. Improved Metabolism of Amino Acids

Upregulation in the processing of amino acid metabolic pathways can occur, such as those of phenylalanine, tyrosine and tryptophan and arginine and proline. This metabolic change to more amino acid metabolism is indicative of greater protein synthesis requirements related to embryonic growth and remodeling of the endometrial development during the early stages of gestation in cattle [72,73]. The metabolism of arginine is of particular importance in the process of reproduction because nitric oxide (NO) is synthesized by the action of nitric oxide synthase (NOS) enzymes on the substrate of arginine [74]. Nitric oxide biosynthesis is important in the process of controlling blood flow to the uterus, embryo growth and implantation [75,76]. The treatment group could have greater NO production through the activation of the mechanism of enhanced arginine metabolism, which contributes to the improvement of uterine perfusion and nutrition of embryos. Also, polyamines are synthesized using arginine, and a lack of polyamines results in cell proliferation and embryonic development [77]. The metabolism of proline is also applicable to reproductive success because proline is a significant constituent of the collagen and proteins of the extracellular matrix [78]. The increased metabolism of proline can contribute to endometrial remodeling and to the changes in structure that the implantation and the placentation require [79,80].

#### 4.4.3. The Downregulation of Inflammatory Lipid Mediators

On day 21 after TAI in the present study, there was an increase in a number of the pro-inflammatory lipid mediators such as gamma-linolenic acid (FC: 1.47, *p*: 0.045), 12(13)-EpOME (FC: 1.51, *p*: 0.013) and conjugated linoleic acids (CLA) and many others were downregulated in the treatment group, including gamma-linolenic acid (FC: 1.47, *p*: 0.04). These results imply that the treatment still has anti-inflammatory effects beyond the essential luteolytic period of MRP and enhances a more conducive uterine milieu to maintain early pregnancy. Gamma-linolenic acid (GLA) is an omega-6 fatty acid polyunsaturated, which can be converted to pro-inflammatory eicosanoids through the arachidonic acid system [81,82]. The finding in the present study indicates that there was a decrease in the GLA concentrations in the cows from the treatment group, which is consistent with the general inhibition of pro-inflammatory lipid metabolism at the end of the treatment period (day 17). Likewise, 12(13)-EpOME (12,13-epoxy-9-octadecenoic acid) is a linoleic acid epoxide which has been linked to inflammatory effects and oxidative stress [83]. So, a reduction in such mediators in the present study may reflect decreased oxidative stress and inflammation.

#### 4.4.4. Alteration in Steroid Hormone Metabolism

On day 21 after TAI of the present study, there was an alteration of steroid hormone metabolites, 5-α-dihydrotestosterone glucuronide (FC: 0.27, *p*: 0.038) downregulation and 3α,20α-dihydroxy-5β-pregnane glucuronide upregulation. These results suggest a change in the metabolism and clearance of steroid hormones in the treatment group. Dihydrotestosterone (DHT) is a potent androgen that is produced through 5α-reduction in testosterone. The DHT glucuronide compound is a conjugated metabolite that results via hepatic glucuronidation, which promotes urinary clearance of DHT [84]. The decrease in the concentration of DHT glucuronides in the treatment group can be attributed to the decrease in androgen metabolism or its retention. Although the functional importance of this change is not directly obvious, there is a possibility that altered androgen metabolism may influence ovarian follicular dynamics, steroidogenesis or endometrial functioning [85]. The 3α,20α-dihydroxy-5β-pregnane glucuronide compound is a conjugated metabolite of progesterone catabolic products [86]. Upregulation in the cows of the hCG+ketoprofen-treated group could be due to a relatively greater metabolism and clearance of progesterone, which could be a secondary effect of relatively greater progesterone production caused by hCG therapy and the anti-luteolytic effect of ketoprofen [87,88].

#### 4.4.5. Kegg Pathway Analysis: Switching to Neuroendocrine and Metabolic Support

The findings from KEGG enrichment analysis at day 21 after TAI revealed that there was a metabolic switch between the direct anti-luteolytic pathways that were active at day 17 after TAI to the long-term reproductive support, which was manifested by the neuroendocrine optimization and the metabolic remodeling. The most evident aspect was the preeminence of tryptophan metabolic processes and serotonergic signaling systems, which were not apparent on day 17 after TAI. This time course indicates that the effects of the treatment change with the progression of the gestational period, with various mechanisms prevailing at different stages. Of special interest is the upregulation of the gap junction and synaptic vesicle cycle pathways, which are normally pathways in neuronal functionality but also in cell–cell communication in non-neuronal tissues [89,90]. Gap junctions are direct cytoplasmic communication channels that are present between neighboring cells and are formed by connexin proteins and are essential in organizing cell activities in the endometrium and corpus luteum [91,92]. Increased gap junction signaling may contribute to greater coordination of endometrial reactions to the embryo and enable coordinated production of progesterone by luteal cells [93]. Overexpression of folate production, cholesterol metabolism and bile acid production may be indicative of a coordinated remodeling of one-carbon and lipid metabolism. These lipid folate pathways are essential for membrane remodeling, methylation processes, steroid metabolism and bile-acid-mediated nutrient signaling [94,95].

### 4.5. Integration of Targeted and Untargeted Metabolomics: A Comprehensive Mechanistic Model

The targeted metabolomics results provide quantitative validation and mechanistic depth to the broader metabolic signatures identified through untargeted profiling. The fact that both methods of analysis led to similar results makes us confident of our proposed mechanisms and indicates that hormonal, inflammatory, oxidative stress and metabolic mechanisms are synchronized to improve pregnancy establishment as a result of the treatment. In particular, results from these targeted analyses provide compelling evidence that the treatment intervention led to the results in the present study of there being the following: (1) relatively greater concentrations of progesterone resulting from hCG-mediated luteal support [96], (2) decreased inflammatory and stress signaling caused by the COX inhibition of ketoprofen and secondary system effects [97] and (3) the establishment of an optimal hormonal milieu with relatively greater progesterone, lesser estradiol and lesser activity of stress hormones [98]. The relatively greater melatonin concentrations, which are indicative of an antioxidant effect [99], indicated by targeted metabolite quantification, provide further insight into the mechanistic interpretation and indicate that the efficacy of the treatment is not confined to the direct pharmacological effect of hCG and ketoprofen but to the stimulation of the endogenous protective mechanisms, which have anti-oxidant, anti-inflammatory and luteal protective functions.

## 5. Conclusions

In conclusion, administering hCG on day 7 after TAI, combined with ketoprofen on days 15 and 16 post-insemination, resulted in the pregnancy percentage in Holstein dairy cows following TAI. Results from metabolomic profiling revealed increases in progesterone and melatonin concentrations and decreases in estradiol and 21-deoxycortisol concentrations, along with suppression of oxidative stress markers and modulation of arachidonic acid, glycerophospholipid and tryptophan metabolism pathways, which are associated with the relatively greater pregnancy percentage in dairy cows.

## Figures and Tables

**Table 1:** Effect of hCG and ketoprofen on pregnancy percentage following TAI.

Groups	No. of Inseminated Cows	No. of Pregnant Cows	Pregnancy Rate (%)
hCG+ketoprofen	278	167	60.07 ^a^
hCG 3 vials/head	268	133	49.63 ^b^
hCG 2 vials/head	253	106	41.90 ^c^

The pregnancy percentages were analyzed using a Chi-square Test. Values with different superscript letters (a–c) differ significantly (*p* < 0.05).

**Table 2:** Fertility-relevant metabolites ranked by biological significance on day 17 subsequent to TAI.

S. No	Metabolite Name	Key Stats
1	sn-Glycero-3-phosphocholine	FC: 2.24, *p*: 0.011, ROC: 1, VIP: 2.29
2	13-OxoODE	FC: 1.39, *p*: 0.037, ROC: 0.92, VIP: 1.58
3	PC(18:0/18:1(9Z)-O(12,13))	FC: 4.74, *p*: 0.019, ROC: 1, VIP: 1.60
4	PC(18:0/18:1(12Z)-O(9S,10R))	FC: 3.44, *p*: 0.033, ROC: 1, VIP:1.65
5	2,3-Dinor-8-iso prostaglandin F2alpha	FC: 1.58, *p*: 0.025, ROC:0.92, VIP: 1.37
6	Arachidonic acid	FC: 2.02, *p*: 0.014, ROC: 0.92, VIP: 1.77
7	LysoPE(P-18:1(9Z)/0:0)	FC: 2.26, *p*: 0.007, ROC: 1, VIP: 1.84
8	phosphoethanolamine (P-18:0)	FC: 2.22, *p*: 0.004, ROC: 1, VIP: 1.72

**Table 3:** Fertility-relevant metabolites ranked by biological significance on day 21 after TAI.

S. No	Metabolite Name	Key Stats
1	LysoPC(22:4)	FC: 0.58, *p*: 0.032, ROC: 0.94, VIP: 2.18
2	Serotonin	FC: 0.72, *p*: 0.038, ROC: 0.83, VIP: 1.74
3	L-Tryptophan	FC: 0.74, *p*: 0.038, ROC: 0.833, VIP: 1.33
4	gamma-Linolenic acid	FC: 1.47, *p*: 0.045, ROC: 0.833, VIP:1.62
5	5-alpha-Dihydrotestosterone glucuronide	FC: 0.27, *p*: 0.038, ROC:0.86, VIP: 1.72
6	12(13)-EpOME	FC: 1.51, *p*: 0.013, ROC: 0.91, VIP: 2.15
7	Conjugated linoleic acids (CLA)	FC: 1.44, *p*: 0.039, ROC: 0.91, VIP: 1.50
8	LysoPE(20:3)	FC: 0.72, *p*: 0.006, ROC: 0.97, VIP: 2.25
9	Glycocholic acid	FC: 0.59, *p*: 0.039, ROC: 0.833, VIP: 1.52

## Data Availability

The original contributions presented in the study are included in the paper, further inquiries can be directed to the corresponding author.

## Supplementary Materials

The following supporting information can be downloaded at: https://www.mdpi.com/article/10.3390/ani16020343/s1. Figure S1: (A,B) KEGG pathway annotation; Figure S2: (A,B) heatmap C4 vs. G4; Figure S3: (A,B) heat map C5 vs. G5.

## References

[1] Ciornei Ș.G. (2021). Embryo Transfer. Animal Reproduction.

[2] Gui L.S., Dai T.S., Guo X.R., Wei S.H., Ma Z.M., Yang D., Ding B., Xiang H., Yu Y., Dan X. (2024). Recent advances in early pregnancy loss diagnosis in dairy cows: New approaches. Reprod. Domest. Anim..

[3] Beasley L., Cogger N., Compton C. (2023). Use of equine chorionic gonadotropin in lactating dairy cattle: A rapid review. N. Z. Vet. J..

[4] Zheng P., Huang H., Li X., Huang F., Adeniran S.O., Wang Z., Feng R., Zhang G. (2021). LRH-A3 and HCG increase pregnancy rate during timed artificial insemination in dairy cows. Anim. Sci. J..

[5] Cunha T.O., Martinez W., Walleser E., Martins J.P.N. (2021). Effects of GnRH and hCG administration during early luteal phase on estrous cycle length, expression of estrus and fertility in lactating dairy cows. Theriogenology.

[6] Santos A., Minela T., Branen J., Pursley J. (2023). Time to increase in pregnancy-specific protein B following artificial insemination is a direct determinant of subsequent pregnancy loss in lactating dairy cows. J. Dairy Sci..

[7] Helmy M.A., Seksaka M.A., Hafez E.M., Elnagar W.M. (2023). Impact of Premature Progesterone Rise on Intracytoplasmic Sperm Injection Outcomes Using Gonadotropin-Releasing Hormone Antagonist Protocol. Zagazig Univ. Med. J..

[8] Hanlon D., Jarratt G., Davidson P., Millar A., Douglas V. (2005). The effect of hCG administration five days after insemination on the first service conception rate of anestrous dairy cows. Theriogenology.

[9] Sánchez J.M., Randi F., Passaro C., Mathew D., Butler S., Lonergan P. (2018). Effect of human chorionic gonadotrophin administration 2 days after insemination on progesterone concentration and pregnancy per artificial insemination in lactating dairy cows. J. Dairy Sci..

[10] Chen F., Hou Ya Zhu X., Mei C., Guo R., Shi Z. (2023). Impact of accessory corpus luteum induced by gonadotropin-releasing hormone or human chorionic gonadotropin on pregnancy rates of dairy cattle following embryo transfer: A META-analysis. Vet. Sci..

[11] Besbaci M., Abdelli A., Minviel J.-J., Belabdi I., Kaidi R., Raboisson D. (2020). Association of pregnancy per artificial insemination with gonadotropin-releasing hormone and human chorionic gonadotropin administered during the luteal phase after artificial insemination in dairy cows: A meta-analysis. J. Dairy Sci..

[12] Bai H., Kawahara M., Takahashi M., Imakawa K. (2022). Recent progress of interferon-tau research and potential direction beyond pregnancy recognition. J. Reprod. Dev..

[13] Sánchez J.M., Mathew D.J., Passaro C., Fair T., Lonergan P. (2018). Embryonic maternal interaction in cattle and its relationship with fertility. Reprod. Domest. Anim..

[14] Wiltbank M.C., Monteiro P.L., Domingues R.R., Andrade J.P.N., Mezera M.A. (2023). Maintenance of the ruminant corpus luteum during pregnancy: Interferon-tau and beyond. Animal.

[15] Binelli M., Thatcher W., Mattos R., Baruselli P.S. (2001). Antiluteolytic strategies to improve fertility in cattle. Theriogenology.

[16] Alkan H., Erdem H. (2018). İneklerde nonsteroid antiinflamatuar ilaçların reprodüktif amaçlı kullanımı. Atatürk Üniv. Vet. Bilim. Derg..

[17] Smith G.W., Davis J.L., Tell L.A., Webb A.I., Riviere J.E. (2008). Extralabel use of nonsteroidal anti-inflammatory drugs in cattle. J. Am. Vet. Med. Assoc..

[18] Bülbül B., Dursun S., Kirbas M., Kose M., Umutlu S. (2010). Düvelerde embriyo transferi öncesi flunixin meglumin uygulamasının gebelik oranı üzerine etkisi. Kafkas Üniv. Vet. Fakültesi Derg..

[19] Gaievski F.R., Kaminski A.P., Kozicki L.E., Segui M.S., Weiss R.R., Bergstein-Galan T.G., Talini R., Valle V.M., Lara N.S.S., Catalano F.A.R. (2024). Comparison of the efficiency of progesterone, ketoprofen, or GnRH administration on the day of fixed-time transfer (FTET) of in vitro produced (IVP) embryos in suckled crossbred beef cows. N. Z. J. Agric. Res..

[20] Dursun Ş. (2011). Laktasyonda Olmayan Isviçre Esmeri Inek ve Düvelerde Ketoprofen ve Flunixin Meglumin Uygulamasının Gebe Kalma Oranı Üzerine Etkisi. Ph.D. Thesis.

[21] Sun H., Wang B., Wang J., Liu H., Liu J. (2016). Biomarker and pathway analyses of urine metabolomics in dairy cows when corn stover replaces alfalfa hay. J. Anim. Sci. Biotechnol..

[22] Kenéz Á., Dänicke S., Rolle-Kampczyk U., von Bergen M., Huber K. (2016). A metabolomics approach to characterize phenotypes of metabolic transition from late pregnancy to early lactation in dairy cows. Metabolomics.

[23] He C., Wang J., Zhang Z., Yang M., Li Y., Tian X., Ma T., Tao J., Zhu K., Song Y. (2016). Mitochondria Synthesize Melatonin to Ameliorate Its Function and Improve Mice Oocyte’s Quality under in Vitro Conditions. Int. J. Mol. Sci..

[24] Uçar E.H., Peker C. (2023). The Effect of Human Chorionic Gonadotropin and Ketoprofen Applications on Pregnancy Rates in Dairy Cows After Artificial Insemination. Anim. Health Prod. Hyg..

[25] Kraevskiy A., Sokolyuk V., Travetskiy M., Chekan O., Musiienko Y. (2020). Surfagon and Ketaprofen for increasing fertility and preventing embryonic death in cows after insemination AY. Ukr. J. Ecol..

[26] Spencer J., Konetchy D., Ahmadzadeh A. (2020). Influences of non-steroidal anti-inflammatory drugs on dairy cattle reproductive performance. Appl. Anim. Sci..

[27] Besbaci M., Abdelli A., Belabdi I., Raboisson D. (2021). Non-steroidal anti-inflammatory drugs at embryo transfer on pregnancy rates in cows: A meta-analysis. Theriogenology.

[28] Sakai S., Yagi M., Fujime N., Kuse M., Sakumoto R., Yamamoto Y., Okuda K., Kimura K. (2021). Heat stress influences the attenuation of prostaglandin synthesis by interferon tau in bovine endometrial cells. Theriogenology.

[29] Garcia-Ispierto I., López-Helguera I., Martino A., López-Gatius F. (2012). Reproductive performance of anoestrous high-producing dairy cows improved by adding equine chorionic gonadotrophin to a progesterone-based oestrous synchronizing protocol. Reprod. Domest. Anim..

[30] Yilmazbas-Mecitoglu G., Gumen A., Karakaya-Bilen E., Keskin A., Guner B., Cakircali R. (2021). The ovulatory response to human chorionic gonadotropin administration on day 4 post timed artificial insemination improved fertility in repeat breeder cows. Acta Vet. Brno.

[31] Wiltbank M., Souza A., Carvalho P., Cunha A., Giordano J., Fricke P., Baez G., Diskin M. (2014). Physiological and practical effects of progesterone on reproduction in dairy cattle. Animal.

[32] Lonergan P., Forde N. (2014). Maternal-embryo interaction leading up to the initiation of implantation of pregnancy in cattle. Animal.

[33] Muñoz M., Uyar A., Correia E., Ponsart C., Guyader-Joly C., Martínez-Bello D., Guienne B.M.-L., Fernandez-Gonzalez A., Díez C., Caamaño J.N. (2014). Metabolomic prediction of pregnancy viability in superovulated cattle embryos and recipients with Fourier transform infrared spectroscopy. BioMed Res. Int..

[34] Aranciaga N., Morton J.D., Berg D.K., Gathercole J.L. (2020). Proteomics and metabolomics in cow fertility: A systematic review. Reproduction.

[35] Boldeanu L., Văduva C.-C., Caragea D.C., Novac M.B., Manasia M., Siloși I., Manolea M.M., Boldeanu M.V., Dijmărescu A.L. (2023). Association between serum 8-iso-prostaglandin F2α as an oxidative stress marker and immunological markers in a cohort of preeclampsia patients. Life.

[36] Yu Y., Yang Q., Wang Z., Ding Q., Li M., Fang Y., He Q., Zhu Y.Z. (2021). The anti-inflammation and anti-nociception effect of ketoprofen in rats could be strengthened through co-delivery of a H_2_S donor, S-propargyl-cysteine. J. Inflamm. Res..

[37] Miyazaki Y., Nakamura T., Takenouchi S., Hayashi A., Omori K., Murata T. (2021). Urinary 8-iso PGF_2α_ and 2,3-dinor-8-iso PGF_2α_ can be indexes of colitis-associated colorectal cancer in mice. PLoS ONE.

[38] Altuna-Coy A., Ruiz-Plazas X., Sánchez-Martin S., Ascaso-Til H., Prados-Saavedra M., Alves-Santiago M., Bernal-Escoté X., Segarra-Tomás J., Chacón M.R. (2022). The lipidomic profile of the tumoral periprostatic adipose tissue reveals alterations in tumor cell’s metabolic crosstalk. BMC Med..

[39] Deluao J.C., Winstanley Y., Robker R.L., Pacella-Ince L., Gonzalez M.B., McPherson N.O. (2022). Oxidative stress and reproductive function: Reactive oxygen species in the mammalian pre-implantation embryo. Reproduction.

[40] Das A., Roychoudhury S. (2022). Reactive oxygen species in the reproductive system: Sources and physiological roles. Oxidative Stress and Toxicity in Reproductive Biology and Medicine: A Comprehensive Update on Male Infertility—Volume One.

[41] Singh S.P., Ankesh Kumar A., Bhavsar P., Bhavsar M., Sourya N., Singh A.K., Sahu M. (2021). Application of non-steroidal anti-inflammatory drugs (NSAIDs) for improvement of cattle fertility. Pharma Innov..

[42] Kmaid S., Albanell S., Saldaña J., Nuñez-Olivera R., Renaud P., Menchaca A. (2025). Pregnancy rate after treatment with a nonsteroidal anti-inflammatory drug (tolfenamic acid) at the time of embryo transfer in recipient cows. Theriogenology.

[43] Reddy G.A.L., Singh M., Kaur R.D. (2023). Modulation of prostaglandin synthesis to improve farm animal reproduction. Int. J. Farm Sci..

[44] Arthiya K. (2022). CRISPR/CAS9 Mediated Editing of Cox-2 Gene in Luteal Cells of Buffalo.

[45] Mahesh G., Anil Kumar K., Reddanna P. (2021). Overview on the discovery and development of anti-inflammatory drugs: Should the focus be on synthesis or degradation of PGE_2_?. J. Inflamm. Res..

[46] Wang B., Wu L., Chen J., Dong L., Chen C., Wen Z., Hu J., Fleming I., Wang D.W. (2021). Metabolism pathways of arachidonic acids: Mechanisms and potential therapeutic targets. Signal Transduct. Target. Ther..

[47] Harwood J.L. (2023). Polyunsaturated fatty acids: Conversion to lipid mediators, roles in inflammatory diseases and dietary sources. Int. J. Mol. Sci..

[48] Gilroy D.W., Bishop-Bailey D. (2019). Lipid mediators in immune regulation and resolution. Br. J. Pharmacol..

[49] Palme R. (2019). Non-invasive measurement of glucocorticoids: Advances and problems. Physiol. Behav..

[50] Penning T.M., Covey D.F. (2024). 5β-Dihydrosteroids: Formation and Properties. Int. J. Mol. Sci..

[51] Wrzecińska M., Czerniawska-Piątkowska E., Kowalczyk A. (2021). The impact of stress and selected environmental factors on cows’ reproduction. J. Appl. Anim. Res..

[52] Fernandez-Novo A., Pérez-Garnelo S.S., Villagrá A., Pérez-Villalobos N., Astiz S. (2020). The effect of stress on reproduction and reproductive technologies in beef cattle—A review. Animals.

[53] Mbiydzenyuy N.E., Qulu L.-A. (2024). Stress, hypothalamic-pituitary-adrenal axis, hypothalamic-pituitary-gonadal axis, and aggression. Metab. Brain Dis..

[54] Dong J., Li J., Li J., Cui L., Meng X., Qu Y., Wang H. (2019). The proliferative effect of cortisol on bovine endometrial epithelial cells. Reprod. Biol. Endocrinol..

[55] Dong J., Qu Y., Li J., Cui L., Wang Y., Lin J., Wang H. (2018). Cortisol inhibits NF-κB and MAPK pathways in LPS activated bovine endometrial epithelial cells. Int. Immunopharmacol..

[56] Cockcroft S. (2021). Mammalian lipids: Structure, synthesis and function. Essays Biochem..

[57] Guardiola S.M.T., Parra C.M., Úbeda C.S. (2024). The Dialogue into the Sow Genital Tract: An Essential Process for Fertility. Assisted Reproductive Technologies in Animals Volume 1: Current Trends for Reproductive Management.

[58] Ridgway N.D. (2021). Phospholipid synthesis in mammalian cells. Biochemistry of Lipids, Lipoproteins and Membranes.

[59] Murakami M., Yamamoto K., Taketomi Y. (2018). Phospholipase A_2_ in skin biology: New insights from gene-manipulated mice and lipidomics. Inflamm. Regen..

[60] Gimeno I., Garcia-Manrique P., Carrocera S., Lopez-Hidalgo C., Valledor L., Martin-Gonzalez D., Gómez E. (2021). The metabolic signature of in vitro produced bovine embryos helps predict pregnancy and birth after embryo transfer. Metabolites.

[61] Gimeno I., García-Manrique P., Carrocera S., López-Hidalgo C., Muñoz M., Valledor L., Martín-González D., Gómez E. (2022). Non-invasive identification of sex in cultured bovine embryos by UHPLC-MS/MS metabolomics. Metabolomics.

[62] Miyamoto A., Shirasuna K. (2018). Luteolysis in the cow: A novel concept of vasoactive molecules. Anim. Reprod. (AR).

[63] Yu X.-H., Tang C.-K. (2022). ABCA1, ABCG1, and cholesterol homeostasis. HDL Metabolism and Diseases.

[64] Wang N., Westerterp M. (2020). ABC transporters, cholesterol efflux, and implications for cardiovascular diseases. Lipid Transfer in Lipoprotein Metabolism and Cardiovascular Disease.

[65] Li D., Tan F., Lin C.S.K., Liu Y., Liu J., Gao C. (2024). Advances in the metabolic engineering of Escherichia coli for the production of serotonin and its precursor, tryptophan. Biochem. Eng. J..

[66] Lv J., Liu F. (2017). The role of serotonin beyond the central nervous system during embryogenesis. Front. Cell. Neurosci..

[67] Amireault P., Sibon D., Côté F. (2013). Life without peripheral serotonin: Insights from tryptophan hydroxylase 1 knockout mice reveal the existence of paracrine/autocrine serotonergic networks. ACS Chem. Neurosci..

[68] Fournier S.B., D’Errico J.N., Stapleton P.A. (2021). Uterine vascular control preconception and during pregnancy. Compr. Physiol..

[69] Grattan D.R., Ladyman S.R. (2024). Maternal Recognition of Pregnancy. Neuroendocrine Regulation of Mammalian Pregnancy and Lactation.

[70] Gumusoglu S., Scroggins S., Vignato J., Santillan D., Santillan M. (2021). The serotonin-immune axis in preeclampsia. Curr. Hypertens. Rep..

[71] Vujovic S., MiomiraIvovic M. (2021). Detection and treatment of some endometrial receptivity disorders–A way to improve fertility rates. Gynecol. Reprod. Endocrinol. Metab..

[72] Gao H. (2020). Amino acids in reproductive nutrition and health. Amino Acids in Nutrition and Health: Amino Acids in Systems Function and Health.

[73] Manta-Vogli P.D., Schulpis K.H., Dotsikas Y., Loukas Y.L. (2020). The significant role of amino acids during pregnancy: Nutritional support. J. Matern.-Fetal Neonatal Med..

[74] Bodis J., Farkas B., Nagy B., Kovacs K., Sulyok E. (2022). The role of L-arginine-NO system in female reproduction: A narrative review. Int. J. Mol. Sci..

[75] Voros C., Sapantzoglou I., Mavrogianni D., Athanasiou D., Varthaliti A., Bananis K., Athanasiou A., Athanasiou A., Papahliou A.M., Zografos C.G. (2025). Unlocking Implantation: The Role of Nitric Oxide, NO_2_-NO_3_, and eNOS in Endometrial Receptivity and IVF Success—A Systematic Review. Int. J. Mol. Sci..

[76] Abdelnaby E.A., Abo El-Maaty A.M. (2021). Melatonin and CIDR improved the follicular and luteal haemodynamics, uterine and ovarian arteries vascular perfusion, ovarian hormones and nitric oxide in cyclic cows. Reprod. Domest. Anim..

[77] Halloran K.M., Stenhouse C., Wu G., Bazer F.W. (2021). Arginine, agmatine, and polyamines: Key regulators of conceptus development in mammals. Amino Acids in Nutrition and Health: Amino Acids in Gene Expression, Metabolic Regulation, and Exercising Performance.

[78] Hu S., He W., Wu G. (2022). Hydroxyproline in animal metabolism, nutrition, and cell signaling. Amino Acids.

[79] Halloran K.M., Stenhouse C. (2025). Key biochemical pathways during pregnancy in livestock: Mechanisms regulating uterine and placental development and function. Reprod. Fertil..

[80] Liu B., Duan L., Liu X., Bazer F.W., Wang X. (2025). Uterine histotroph and conceptus development. IV. Metabolomic analyses of uterine luminal fluid reveals regulatory landscapes during the peri-implantation period of pregnancy in pigs. Biol. Reprod..

[81] Hegazy M.A., Ahmed S.M., Sultan S.M., Afifi O.F., Mohamed M.A., Azab A.E., Hassanen M.A., Zaben R.K. (2025). Metabolic dysfunction-associated steatotic liver disease and omega-6 polyunsaturated fatty acids: Friends or foes. World J. Hepatol..

[82] Xu R., Molenaar A.J., Chen Z., Yuan Y. (2025). Mode and Mechanism of Action of Omega-3 and Omega-6 Unsaturated Fatty Acids in Chronic Diseases. Nutrients.

[83] Anton P., Rutt L.N., Capper C., McCullough R. (2023). Profiling the Oxylipidome in Aged Mice after Chronic Ethanol Feeding: Identifying a Disconnect between Cytokines and Lipid Metabolites. SSRN Electron. J..

[84] Tsiara I., Correia M.S., Yang F., Zeng W., Seeburger P., Hervás Povo B., Lundgaard I., Menéndez-González M., Vujasinovic M., Löhr J.-M. (2025). Global metabolomics profiling of glucuronides in human plasma, fecal, and cerebrospinal fluid samples. Analytical and Bioanalytical Chemistry.

[85] Xu X., Bai J., Liu K., Xiao L., Qin Y., Gao M., Liu Y. (2023). Association of metabolic and endocrine disorders with bovine ovarian follicular cysts. Animals.

[86] Ku C.W., Tan Z.W., Lim M.K., Tam Z.Y., Lin C.-H., Ng S.P., Allen J.C., Lek S.M., Tan T.C., Tan N.S. (2017). Spontaneous miscarriage in first trimester pregnancy is associated with altered urinary metabolite profile. BBA Clin..

[87] Dorfner M., Klein J., Senkleiter K., Lanig H., Kreis W., Munkert J. (2024). Addressing the Evolution of Cardenolide Formation in Iridoid-Synthesizing Plants: Site-Directed Mutagenesis of PRISEs (Progesterone-5β-Reductase/Iridoid Synthase-like Enzymes) of Plantago Species. Molecules.

[88] Bäckström T., Andreen L., Birzniece V., Björn I., Johansson I.-M., Nordenstam-Haghjo M., Nyberg S., Sundström-Poromaa I., Wahlström G., Wang M. (2003). The role of hormones and hormonal treatments in premenstrual syndrome. CNS Drugs.

[89] Schiera G., Di Liegro C.M., Di Liegro I. (2019). Cell-to-cell communication in learning and memory: From neuro-and glio-transmission to information exchange mediated by extracellular vesicles. Int. J. Mol. Sci..

[90] Pacwa A., Mroz K., Liu X., Smedowski A. (2025). The structure, function, and distribution of gap junctions in the retina: Life cycle in health and disease. J. Cell Commun. Signal..

[91] Kibschull M., Gellhaus A., Carette D., Segretain D., Pointis G., Gilleron J. (2015). Physiological roles of connexins and pannexins in reproductive organs. Cell. Mol. Life Sci..

[92] Marchais M., Gilbert I., Bastien A., Macaulay A., Robert C. (2022). Mammalian cumulus-oocyte complex communication: A dialog through long and short distance messaging. J. Assist. Reprod. Genet..

[93] Kshersagar J., Damle M.N., Sharma R., Joshi M.G. (2025). Cell Communication in Endometrium: Understanding and Improving Endometrial Biomarkers. Cell Biology and Translational Medicine.

[94] Ducker G.S., Rabinowitz J.D. (2017). One-carbon metabolism in health and disease. Cell Metab..

[95] Scheyer N., Guéant-Rodriguez R.-M., Agopiantz M., Leininger-Muller B. (2025). Steroids and One-Carbon Metabolism: Clinical Implications in Endocrine Disorders. Neuroendocrinology.

[96] Qi L., Liu Y., Mei H., Mao H., Chen L., Wang A., Dai Z., Weng S., Wang M., Ke Z. (2025). Heterodimeric Fc fused porcine FSH improved the reproductive performance of gilts. Theriogenology.

[97] Cui L., Guo J., Wang Z., Zhang J., Li W., Dong J., Liu K., Guo L., Li J., Wang H. (2023). Meloxicam inhibited oxidative stress and inflammatory response of LPS-stimulated bovine endometrial epithelial cells through Nrf2 and NF-κB pathways. Int. Immunopharmacol..

[98] Pereira G., Guo Y., Silva E., Bevilacqua C., Charpigny G., Lopes-da-Costa L., Humblot P. (2022). Progesterone differentially affects the transcriptomic profiles of cow endometrial cell types. BMC Genom..

[99] Gutiérrez-Añez J.C., Henning H., Lucas-Hahn A., Baulain U., Aldag P., Sieg B., Hensel V., Herrmann D., Niemann H. (2021). Melatonin improves rate of monospermic fertilization and early embryo development in a bovine IVF system. PLoS ONE.

