# Supplementary figures and images for: Metabolomic Markers Reveal How hCG–Ketoprofen Intervention Increase Pregnancy Percentage Following Timed Artificial Insemination in Dairy Cows
Source: Animals (Basel). 2026 Jan 22;16(2):343. doi: 10.3390/ani16020343 (PMC12837658; doi:10.3390/ani16020343)

S1 A

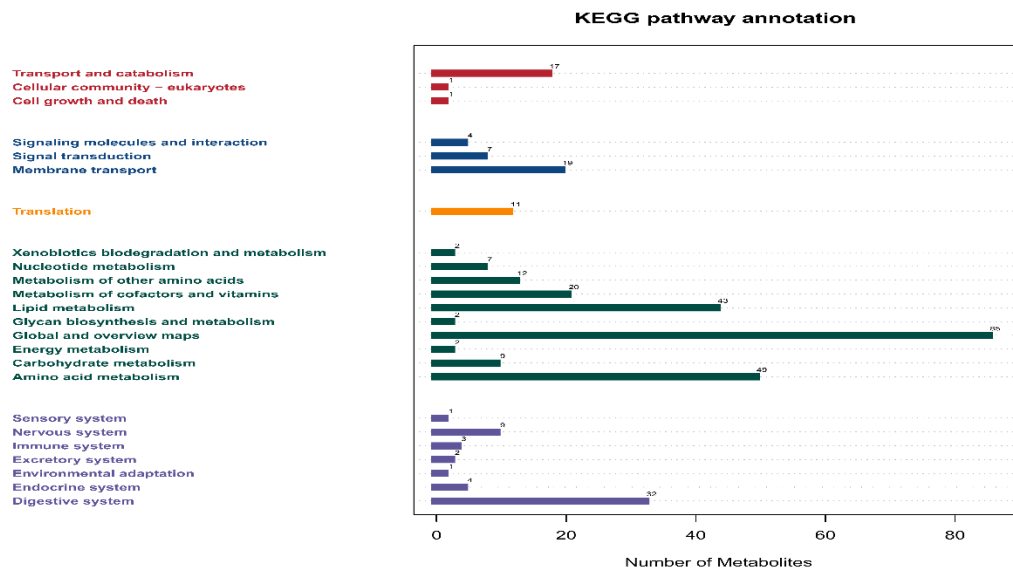

S1 B

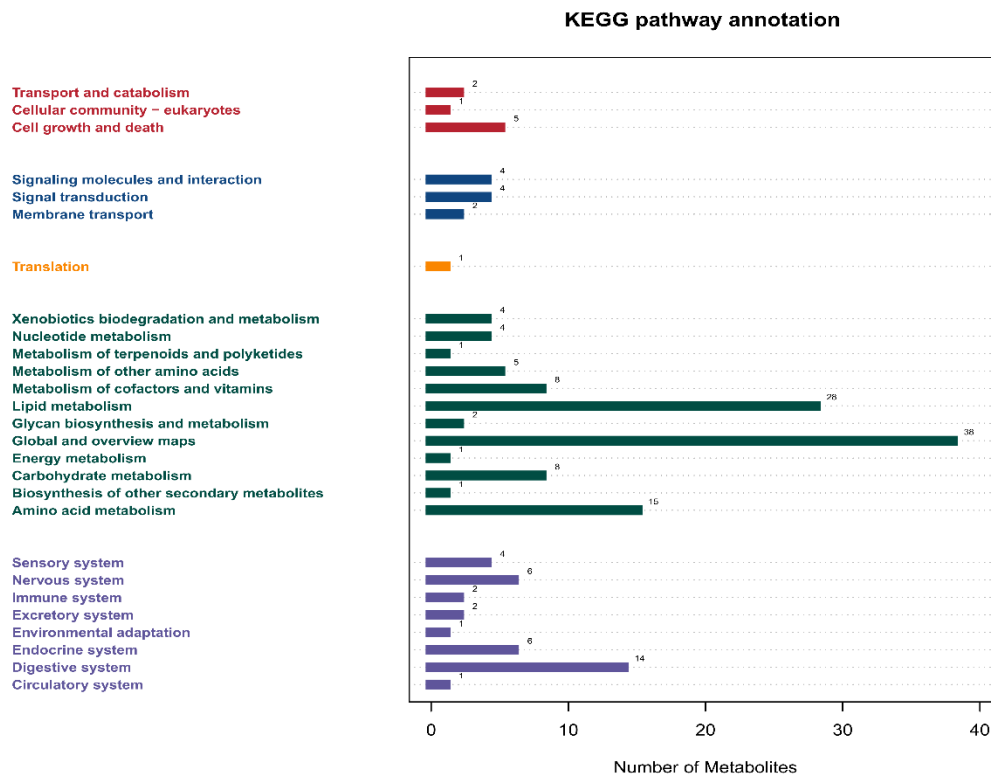

S2 A

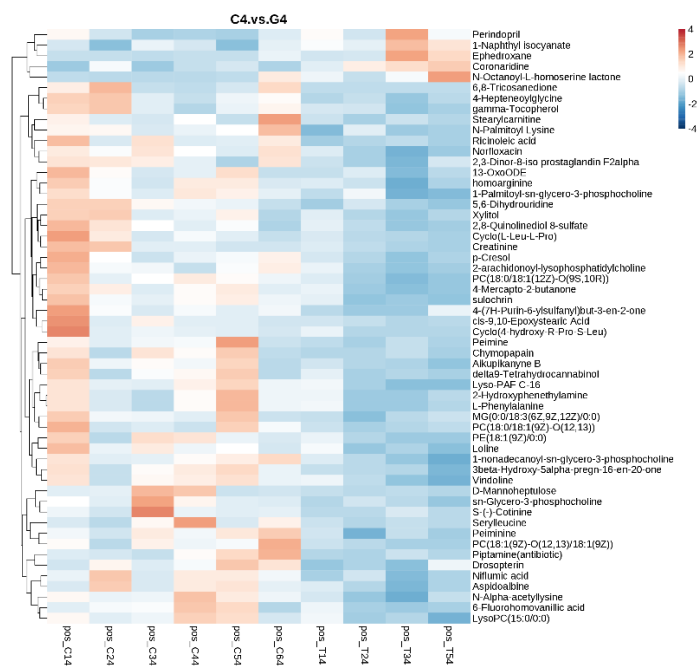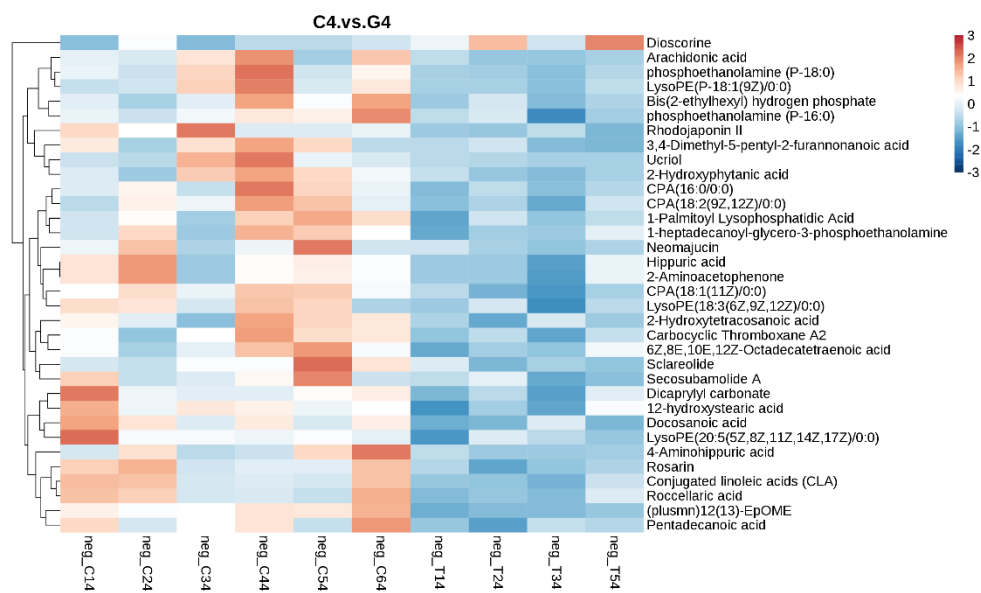

S2 B

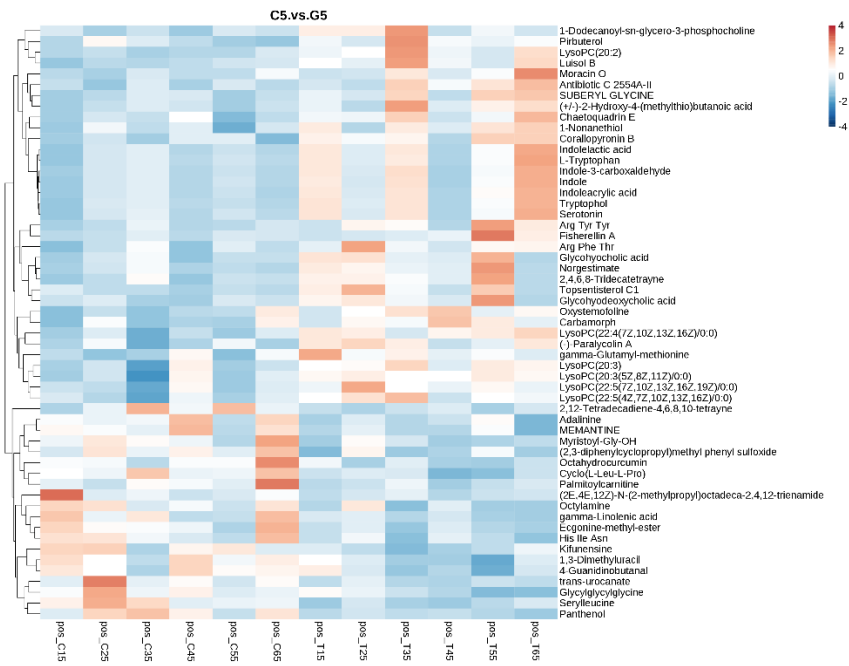

### S3 A

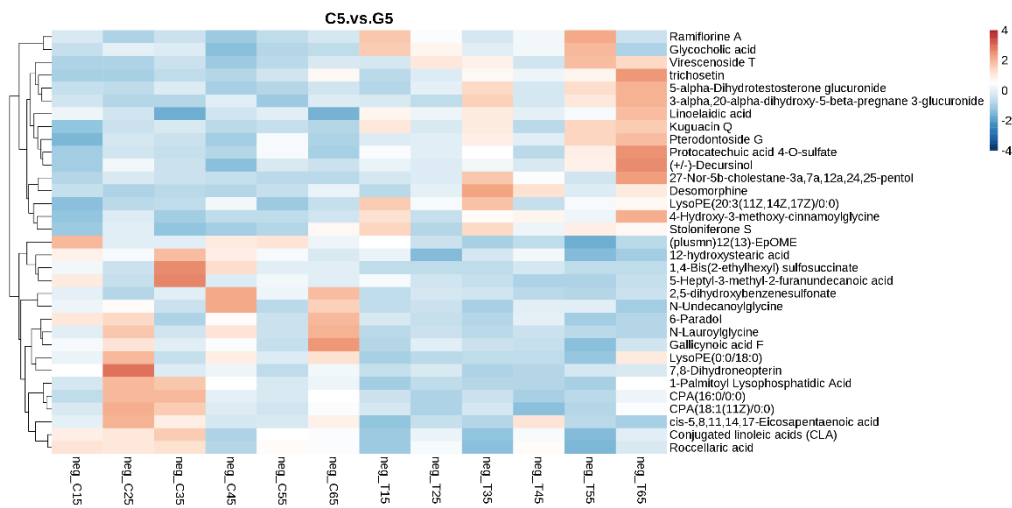

### S3 B

Supplement: Supplementary file 1 [file animals-16-00343-s001.zip › animals-4073126-supplementary.pdf]
